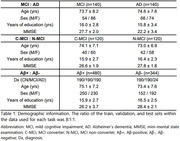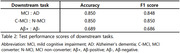# Latent Representation Learning of Cortical Atrophy using BERT‐based Foundation Model

**DOI:** 10.1002/alz.090039

**Published:** 2025-01-09

**Authors:** Heeyun An, Jiyun Kim, Joon‐Kyung Seong, Wha Jin Lee

**Affiliations:** ^1^ NeuroXT, Seoul Korea, Republic of (South); ^2^ Korea University, Seoul Korea, Republic of (South)

## Abstract

**Background:**

The assessment of cortical thickness through structural magnetic resonance imaging (MRI) reveals various cortical atrophy patterns, delivering crucial insights into the extent of neurodegeneration. Numerous studies have focused on these thickness values to construct models for diverse neurodegeneration‐related tasks, such as classifying disease groups, staging progression, or predicting progression speed. However, these tasks require substantial training datasets tailored to each specific task, necessitating the development of new task‐specific models. We aimed to establish a foundational model using pre‐trained BERT, integrating context‐aware deep latent representations derived from a large cohort of unlabeled cortical atrophy data, to address these challenges.

**Method:**

We collected 10,450 T1‐weighted MRI images within the ADNI dataset. Cortical thickness was derived using FreeSurfer across 68 regions based on the Desikan‐Killiany atlas. Emplying the BERT model, we applied its self‐attention capabilities to encode deep bidrectional representations of the data. The model is pre‐trained with 8,360 unlabeled cortical thickness data through self‐supervised learning, predicting randomly masked thickness values. Subsequently, we fine‐tuned this pre‐trained model with only one additional output layer using a supervised approach, employing independent data subsets for three challenging downstream classification tasks: (1) mild cognitive impairment (MCI) and Alzheimer’s dementia (AD), (2) MCI converters (C‐MCI) and non‐converters (N‐MCI) within 3 years, and (3) Aβ‐positive and negative cases. During fine‐tuning, demographic information encoded by one layer was also incorporated for classification purposes.

**Result:**

Table 1 provides demographic details of the subset data for fine‐tuning, showing comparable trends in each set of information, except for MMSE in the MCI and AD comparison. Table 2 outlines our model's performance on three downstream classification tasks. Despite training with a relatively limited amount of data, our model demonstrated competitive performance with state‐of‐the‐art methods, achieving 85% accuracy on the classification of MCI and AD, 85% of C‐MCI and N‐MCI, and 69% of Aβ‐positive and negative.

**Conclusion:**

This study introduces the novel cortical atrophy foundation model, demonstrating competitive performance even with a limited dataset across diverse challenging tasks. The model's performance in predictive tasks highlights its potential applicability for various nerodegeneration‐related challenges, paving the way for advanced AI applications within the realm of neurodegenerative diseases.